# Narrative review of mitochondrial dysfunction in aging-related salt-sensitive hypertension: outcomes, mechanisms, and therapeutic implications

**DOI:** 10.4103/agingadv.agingadv-d-25-00018

**Published:** 2025-11-24

**Authors:** Sepiso K. Masenga, Joreen P. Povia, Bislom C. Mweene, Ronald McMillian, Claude Albritton, Taneisha Gillyard, Jeremiah Afolabi, Edgar Garza Lopez, Benjamin Rodriguez, Amber Crabtree, Salma AshShareef, Margaret Mungai, Han Le, Andrea Marshall, Prasanna Katti, Kit Neikirk, Annet Kirabo, Antentor Hinton

**Affiliations:** 1 Department of Cardiovascular Science and Metabolic Diseases, Livingstone Center for Prevention and Translational Science, Livingstone, Zambia;; 2 Department of Medicine, Division of Clinical Pharmacology, Vanderbilt University Medical Center, Nashville, TN, USA;; 3 Department of Molecular Physiology & Biophysics, Vanderbilt University, Nashville, TN, USA;; 4 Department of Biomedical Sciences, Meharry Medical College, Nashville, TN, USA;; 5 Department of Biology, Indian Institute of Science Education and Research (IISER), Tirupati, AP, India;; 6 The Center for AIDS Health Disparities Research, Meharry Medical College, Nashville, TN, USA.;; 7 Department of Microbiology, Immunology, and Physiology, Meharry Medical College, Nashville, TN, USA;; 8 Department of Biochemistry, Cancer Biology, Pharmacology, and Neuroscience, Meharry Medical College, Nashville, TN, USA;; 9 Vanderbilt Center for Immunobiology, Vanderbilt Institute for Infection, Immunology and Inflammation, Vanderbilt Institute for Global Health, Vanderbilt University Medical Center, Nashville, TN, USA

**Keywords:** aging, cristae remodeling, fission, fusion, mitochondria-endoplasmic reticulum contact, mitochondrial dynamics, mitochondrial dysfunction, mitochondrial-targeted therapies, oxidative stress, salt-sensitive hypertension

## Abstract

Salt sensitivity of blood pressure is prevalent in the aging population, characterized by an exaggerated hypertensive response to dietary sodium intake. Emerging evidence implicates mitochondrial dysfunction as a central contributor to salt sensitivity of blood pressure with mechanistic involvement of oxidative stress, endoplasmic reticulum stress, disrupted mitochondrial-endoplasmic reticulum contacts, and impaired autophagy. This review explores the interplay between aging, mitochondrial dysfunction, and salt sensitivity of blood pressure. Morphological mitochondrial changes including mitochondrial fragmentation due to fission-fusion imbalances, cristae remodeling leading to bioenergetic deficits, and mitochondrial-endoplasmic reticulum contact disruptions affecting calcium homeostasis across aging are contextualized in salt sensitivity of blood pressure. Alongside these changes, age-associated impairments in mitophagy result in the accumulation of defective mitochondria, exacerbating oxidative stress and inflammation. Understanding these pathways offers potential therapeutic avenues to attenuate salt sensitivity of blood pressure in older adults.

## Editor note

AHJ is an Editorial Board Member of Aging Advances. The article was subject to the journal’s standard procedures, with peer review handled independently of this Editorial Board Member and their research groups.

## Introduction

Hypertension remains a leading modifiable risk factor for cardiovascular and renal disease, and according to the World Health Organization, hypertension affects over 1.3 billion people globally.^1^ Its prevalence rises sharply with advancing age: in adults over 60 years old, more than 60% of who are hypertensive.^2,3^ This age-related surge in hypertension contributes substantially to healthcare burdens, owing to elevated risk of stroke, myocardial infarction, heart failure, and kidney disease.^4,5^ A particularly important phenotype in the elderly is salt sensitivity of blood pressure (SSBP), an exaggerated blood pressure response to dietary sodium, which is observed in an estimated one-third of healthy individuals and over half of those with established hypertension.^6,7^ SSBP is more prevalent in older adults and constitutes an independent predictor of cardiovascular morbidity and mortality.^6,7^ The aging population, therefore, experiences both a high prevalence of hypertension and an increased likelihood of salt-sensitive hypertension, which together complicates blood pressure management and raises the risk of organ damage.^6,8^ These epidemiological trends underscore the urgency of elucidating mechanisms behind aging-related SSBP and developing targeted interventions.

The mechanisms driving SSBP remain incompletely understood, though mitochondrial dysfunction has emerged as a pivotal player.^9^ Mitochondria interact with the endoplasmic reticulum (ER) to regulate cellular energy and redox balance, affecting nitric oxide levels, vascular tone, and sodium transport in vascular and renal cells.^10,11^ Furthermore, aging exacerbates mitochondrial and ER stress, causes mitochondrial DNA damage, reduces biogenesis, imbalances fission-fusion, decreases efficiency, increases reactive oxygen species (ROS) generation, impairs autophagic clearance, and disrupts mitochondrial-endoplasmic reticulum contacts (MERCs), which collectively blunt renal sodium excretion and stiffen blood vessels, promoting salt-sensitive hypertension.^9,12,13^

In aggregate, the confluence of high salt intake with age-impaired mitochondrial pathways sets the stage for SSBP. Understanding the interplay between aging, mitochondrial dysfunction, and salt-sensitive hypertension is not only mechanistically illuminating but also of high clinical importance. Despite this importance, few studies that have attempted to link SSBP to mitochondrial dysfunction and aging. Recently, Demirci et al.^8^ showed aging, inflammation, and sex differences interact within SSBP but do not fully explore mitochondrial energetics in blood pressure regulation. While sex differences in SSBP are acknowledged,^14^ the influence of mitochondrial function on these differences is poorly characterized. Given that mitochondria are influenced by sex hormones,^15^ further research is needed to explore how these hormones modulate mitochondrial efficiency and oxidative stress in relation to SSBP. Therefore, a critical gap remains in understanding how mitochondrial oxidative phosphorylation deficits, adenosine triphosphate (ATP) depletion, and excessive ROS generation alter renal sodium handling. This review explores the interplay between aging, salt-sensitive hypertension, and mitochondrial function, highlighting underlying mechanisms and signal transduction pathways that mediate these effects.

## Search Strategy

For this review, we performed comprehensive literature searches in PubMed and Web of Science for English-language articles up to May 2025. The following keywords and MeSH terms were used in various combinations: “salt-sensitive hypertension,” “aging” or “age-related,” “mitochondrial dysfunction,” “oxidative stress,” “mitochondria-associated membranes,” “MAMs,” “mitochondria-endoplasmic reticulum contact sites,” “MERCs,” “mitophagy,” “autophagy,” and “blood pressure.” We focused on publications from the past ~20 years (2005–2025) to capture recent advances, but also included seminal older studies that established fundamental concepts of salt sensitivity and mitochondrial biology. Additional sources were identified by screening the references of relevant papers, including recent review articles. Studies were selected for inclusion if they addressed mechanisms linking dietary salt, blood pressure, and mitochondria in either animal models or clinical research or if they evaluated therapeutic interventions targeting mitochondrial pathways in hypertension. Both original research articles and high-impact reviews were included to ensure a balanced and up-to-date overview. Our search strategy yielded a broad scope of evidence spanning molecular signaling, animal models of salt-sensitive hypertension, and emerging clinical insights, which together inform the narrative of this review.

## Hypertension as a Mitochondrial Disease

Hypertension is increasingly recognized as a disorder of mitochondrial dysfunction, characterized by oxidative stress, impaired calcium handling, and disrupted biogenesis/dynamics in the kidney, heart, and vasculature.^16–18^ Hypertension induces mitochondrial fragmentation, cristae loss, and swelling in mitochondrial size that drive organ dysfunction through bioenergetic failure, oxidative stress, and apoptosis,^19^ processes that are worsened with aging.^20^ Together this results in profound dysregulation in hypertensive states, disrupting their roles in energy production and cellular homeostasis, contributing to vascular, renal, and cardiac pathology.^21^ While mitochondrial are multifaceted in both function and mechanisms of quality control,^22,23^ aging displays several hallmarks including a decline in mitochondrial biogenesis (via peroxisome proliferator-activated receptor gamma coactivator 1-alpha [PGC1α] and nuclear respiratory factors 1 and 2 [NRF1/2]), maintenance of mRNA (through mitochondrial transcription factor A [TFAM]) and in autophagy, together exacerbating bioenergetic deficits, promoting SSBP.^24^ Additionally, with aging, redox status is altered, wherein mitochondrial ROS damages DNA, lipids, and proteins, impairing vascular contractility and renal sodium excretion.^25^ Finally, downregulation of fusion proteins (mitofusins [MFN2], optic atrophy 1 [OPA1]) and upregulation of fission mediators (dynamin-related protein 1 [Drp1] and its recruitment partner mitochondrial fission factor [Mff]) reduce oxidative capacity.^26,27^

While these concomitant actions occur across aging, it is important to realize they often have confluent effects, causing positive feedback loops to exacerbate renal dysfunction. Both MFN2, a mitochondria protein regulating the structure and function of mitochondria, and OPA1, a protein regulating mitochondrial dynamics, are downregulated in hypertension, reducing mitochondrial fusion and compromising oxidative capacity.^26,27^ MFN2 loss in vascular smooth muscle cells (VSMCs) exacerbates ROS production and endothelial dysfunction. Excessive mitochondrial ROS production in these tissues damages DNA, lipids, and proteins, while dysregulated calcium fluxes impair vascular smooth muscle contractility and renal sodium excretion.^25^ Additionally, aging-related declines in mitochondrial biogenesis (via PGC1α/NRF1/2 pathways) and fission-fusion imbalance exacerbate bioenergetic deficits, promoting endothelial dysfunction and salt-sensitive hypertension.^24^ These mitochondrial anomalies align with the structural and functional roles of mitochondrial contact site and cristae organizing system (MICOS), a multi-protein complex that supports cristae junctions formation and maintenance,^28^ suggesting its potential involvement in hypertension progression. A summary of mitochondria dynamics in hypertension is presented in Table 1. These interconnected mitochondrial abnormalities underscore their central role in the pathogenesis of hypertension and highlight mitochondrial structure–function disruption as a critical target in aging-related salt-sensitive hypertension.

### Beyond hypertension

Mitochondria dynamic alterations have been implicated in many hypertension-related adverse outcomes including cardiovascular diseases such as heart failure.^17,18^ For example, In the early stages of heart failure, mitochondrial dynamics shift as levels of the outer-membrane fusion proteins MFN1 and MFN2 fall while the fission GTPase DRP1 rises, pushing the balance toward fragmentation^29–33^; loss of either MFN paralogue or both produces fragmented mitochondria with patchy membrane potential and weak respiration, changes that foreshadow contractile decline.^29–33^ As disease progresses, DRP1-driven fission becomes excessive as evidenced in pulmonary-hypertension models, lowering ATP output and worsening pump function, yet partial inhibition of DRP1 can restore energy production and improve ejection fraction, whereas complete DRP1 ablation provokes dilated cardiomyopathy, underscoring the need for a calibrated fusion-fission ratio.^34–36^ Inner-membrane remodeling accompanies these events: the fusion GTPase OPA1 is post-transcriptionally depleted in failing hearts, and stress-activated proteases YME1L (under basal conditions) and OMA1 (under severe stress) cleave it into short forms that collapse cristae and blunt oxidative phosphorylation; excessive OPA1 processing induces fragmentation and apoptosis, but loss of OMA1 preserves cristae and protects the ventricle.^37–43^ OMA1 activation also triggers a DELE1-ATF4 stress pathway that feeds back on OPA1-mediated contact-site formation, further tying mitochondrial structure to integrated stress signaling.^44–46^ DRP1 activity itself is modulated by post-translational marks: phosphorylation at S616 or S693 stimulates fission, while phosphorylation at S637 or S656 restrains it; calcineurin-mediated dephosphorylation of S637 during ischemia–reperfusion tilts the heart toward excessive fission and dysfunction.^47,48^ In the late stages of heart failure, adaptor proteins which support fusion and fission (e.g., mitochondrial fission factor [MFF], mitochondrial fission 1 [FIS1], and mitochondrial dynamics 49 and 51 [MiD49/51]) together with modifiers like PGAM5, BNIP3, Prohibitin-2, and TMEM135, fine-tune or disrupt the fusion-fission equilibrium, altering fatty-acid oxidation, ER stress, and overall mitochondrial quality.^49–52^ Taken together, heart failure unfolds through a chronological cascade: initial MFN/DRP1 imbalance, subsequent OPA1 cleavage and crista collapse, and finally diverse adaptor-protein and post-translational changes that collectively remodel mitochondrial networks, undermine bioenergetics, and drive cellular demise, implying that therapeutic success will hinge on restoring, rather than abolishing, the heart’s dynamic mitochondrial balance.

Altogether, these findings highlight that progressive heart failure is tightly linked to stage-specific disruptions in mitochondrial dynamics, emphasizing that therapeutic strategies must aim to restore, not eliminate, the delicate balance between mitochondrial fusion and fission.

### Mitochondrial–nuclear communication in hypertension

Mitochondria-nucleus crosstalk regulates key processes disrupted in hypertension, including metabolic adaptation, cell survival, and inflammatory responses.^53^ MICOS-mediated cristae integrity ensures efficient electron transport chain (ETC) function, which influences retrograde signaling pathways such as activating transcription factor 4 (ATF4) and C/EBP Homologous Protein (CHOP) activation under stress.^25^ Disrupted MICOS could impair mitochondrial membrane potential, triggering ROS overproduction and calcium mishandling, thereby activating pro-inflammatory and pro-fibrotic nuclear transcription factors (e.g., nuclear factor kappa-B).^16^ This bidirectional communication underscores how mitochondrial structural defects can propagate systemic hypertension.

Few studies have directly investigated MICOS in hypertension, and none have utilized 3D imaging to explore mito–nuclear crosstalk.^54^ The studies done by the Hinton laboratory^54^ pioneer the use of advanced imaging to visualize MICOSdependent cristae remodeling and its impact on nuclear transcriptional programs in hypertensive models, offering unprecedented spatial resolution of organelle interactions.

### Mechanistic and therapeutic implications of mitochondrial contact site and cristae organizing system dysfunction

Collectively, MICOS dysfunction disrupts mitochondrial architecture and signaling, amplifying oxidative stress, impairing energy production, and promoting maladaptive vascular and renal remodeling in hypertension. This can include MICOS destabilization impairing ETC efficiency, lowering ATP synthesis, exacerbating bioenergetic failure in vascular and renal cells.^24^ Additionally, impaired MICOS can dysregulate ATF4 and CHOP, mediators of the mitochondrial unfolded protein response, while aberrant calcium flux may activate calcineurin/NFAT pathways, driving vascular remodeling.^53^ Concurrently, MICOS dysfunction may downregulate PGC1α and NRF1/2, reducing mitochondrial biogenesis and antioxidant defenses, thereby perpetuating oxidative stress.^25^ Given these multifaceted roles of the MICOS complex remain poorly elucidated in the context of SSBP, yet restoring MICOS expression or structure could mitigate hypertensive phenotypes. CRISPRa/dCas9 strategies to upregulate MIC60/MIC19 or small molecules like SS-31 that stabilize cristae architecture may improve mitochondrial function.^16^ Compounds targeting MICOS-associated proteins (e.g., SAMM50) are under exploration to enhance cristae integrity and ATP production.^23^ These emerging strategies suggest that therapeutic restoration of MICOS holds promise for reversing mitochondrial dysfunction and alleviating hypertension-related organ damage.

### Mitochondrial contact site and cristae organizing system, mitochondrial-derived vesicles, and mitochondrial–endoplasmic reticulum contacts in hypertension

MICOS dysfunction may alter mitochondrial-derived vesicle formation, impairing quality control and exacerbating inflammasome activation in hypertensive cells.^24^ Additionally, MICOS interacts with MERCs, which regulate calcium and ROS signaling. Disrupted MICOS-MERCs crosstalk could amplify ER stress and cytosolic calcium overload, both of which are key contributors to vascular dysfunction.^25^ Targeting these interfaces may offer novel therapeutic avenues.

## Mechanisms of Mitochondrial Dysfunction in Salt-Sensitive Hypertension

### Mitochondrial fragmentation and fission–fusion imbalance

**Figure 1 |:**
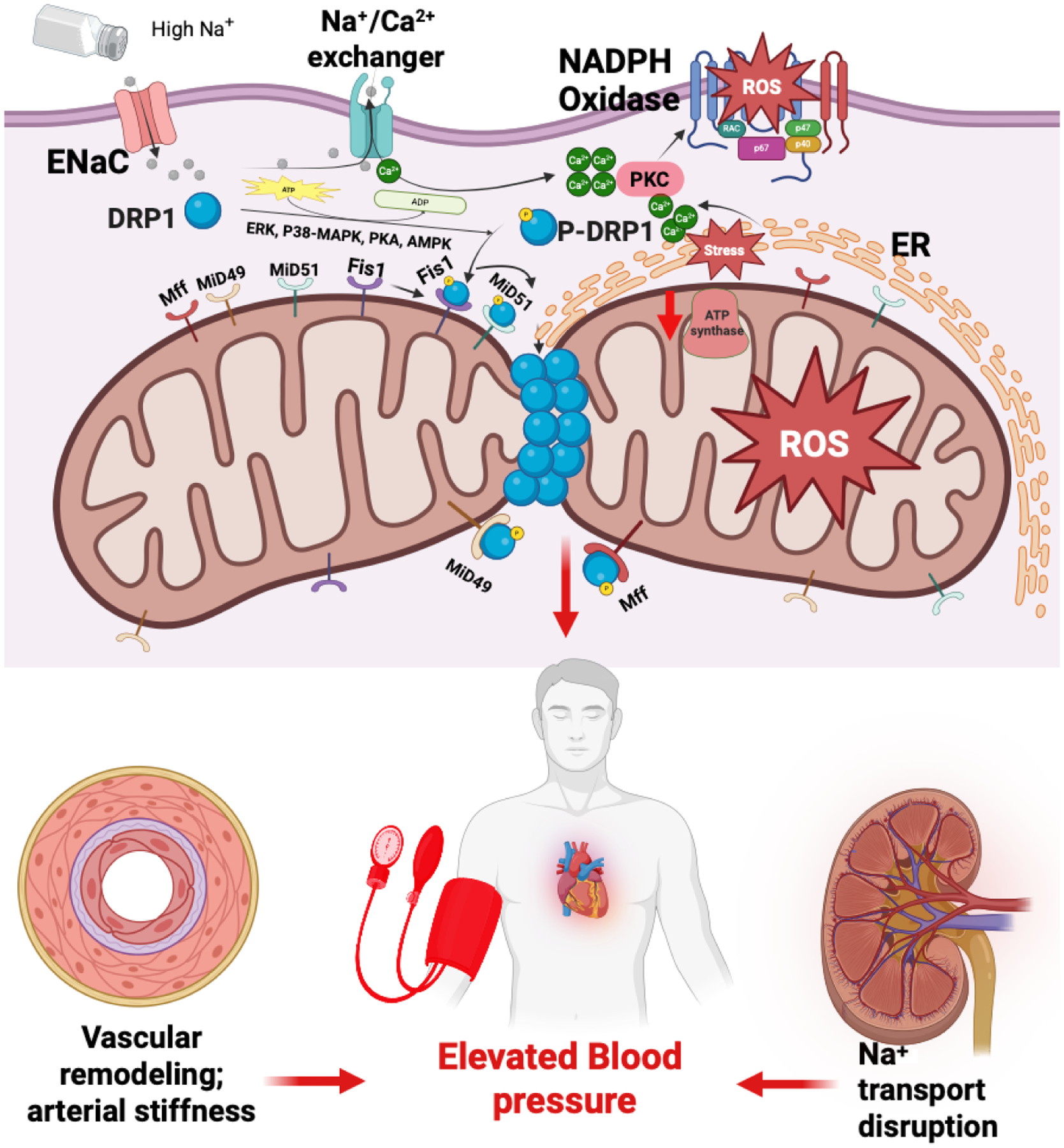
Mitochondrial fission in salt sensitivity of blood pressure (SSBP). A diagram illustrating DRP1-mediated mitochondrial fission leading to fragmented mitochondria, increased reactive oxygen species (ROS), and reduced adenosine triphosphate (ATP) production. High salt intake enhances epithelial sodium channel (ENaC) activity, facilitating Na+ entry into endothelial cells or myocytes. This sodium influx may lead to increased Ca^2+^ entry, activating protein kinase C (PKC). Subsequently, PKC activation could result in elevated ROS production and endoplasmic reticulum (ER) stress, which may enhance DRP1-driven mitochondrial fission. This sequence of events could reduce ATP production in myocardial cells, potentially exacerbating hypertension. Created with BioRender.com. ADP: adenosine diphosphate; AMPK: AMP-activated protein kinase; Erk: extracellular signal-regulated kinase; Fis1: mitochondrial fission 1 protein; Mff: mitochondrial fission factor; Mid49: mitochondrial dynamics protein of 49 kDa; Mid51: mitochondrial dynamics protein of 51 kDa; P-DRP1: phosphorylated dynamin-related protein 1; PKA: protein kinase A; p38-MAPK: p38 mitogen-activated protein kinase.

Mitochondrial structure is regulated by fission (fragmentation) and fusion (elongation). Drp1 activation is linked to renal mitochondrial fragmentation and podocyte injury in salt-sensitive hypertension.^55^ In salt-sensitive hypertension, excessive mitochondrial fission mediated by Drp1 occurs in renal and vascular tissues, leading to fragmented mitochondria (Figure 1). This fragmentation reduces ATP production and increases ROS and mitochondrial DNA damage. These disruptions impair sodium transport in renal tubules, promote endothelial dysfunction, and exacerbate vascular stiffness, worsening hypertension.^56^ The initial ER–mitochondria contact initiates marking of the fission points upon which the activated DRP1 will localize to initiate fission via mitochondria adaptor proteins, including Mff, Mid49/Mid51, and Fis1.^57^ Research supports the role of Drp1-mediated mitochondrial fission in cardiovascular diseases. For instance, increased Drp1 activity has been linked to mitochondrial fragmentation, elevated ROS levels, and reduced ATP production in myocardial cells, contributing to cardiac dysfunction.^58^ Additionally, studies have shown that factors like high glucose levels can induce Drp1-mediated mitochondrial fission via calcium influx, leading to cardiomyocyte hypertrophy.^59^ These findings align with our hypothesis that high salt-induced ENaC activity may trigger a similar pathway, culminating in Drp1-mediated mitochondrial fission and impaired cardiac energy metabolism.

### Cristae remodeling and bioenergetic dysfunction

**Figure 2 |:**
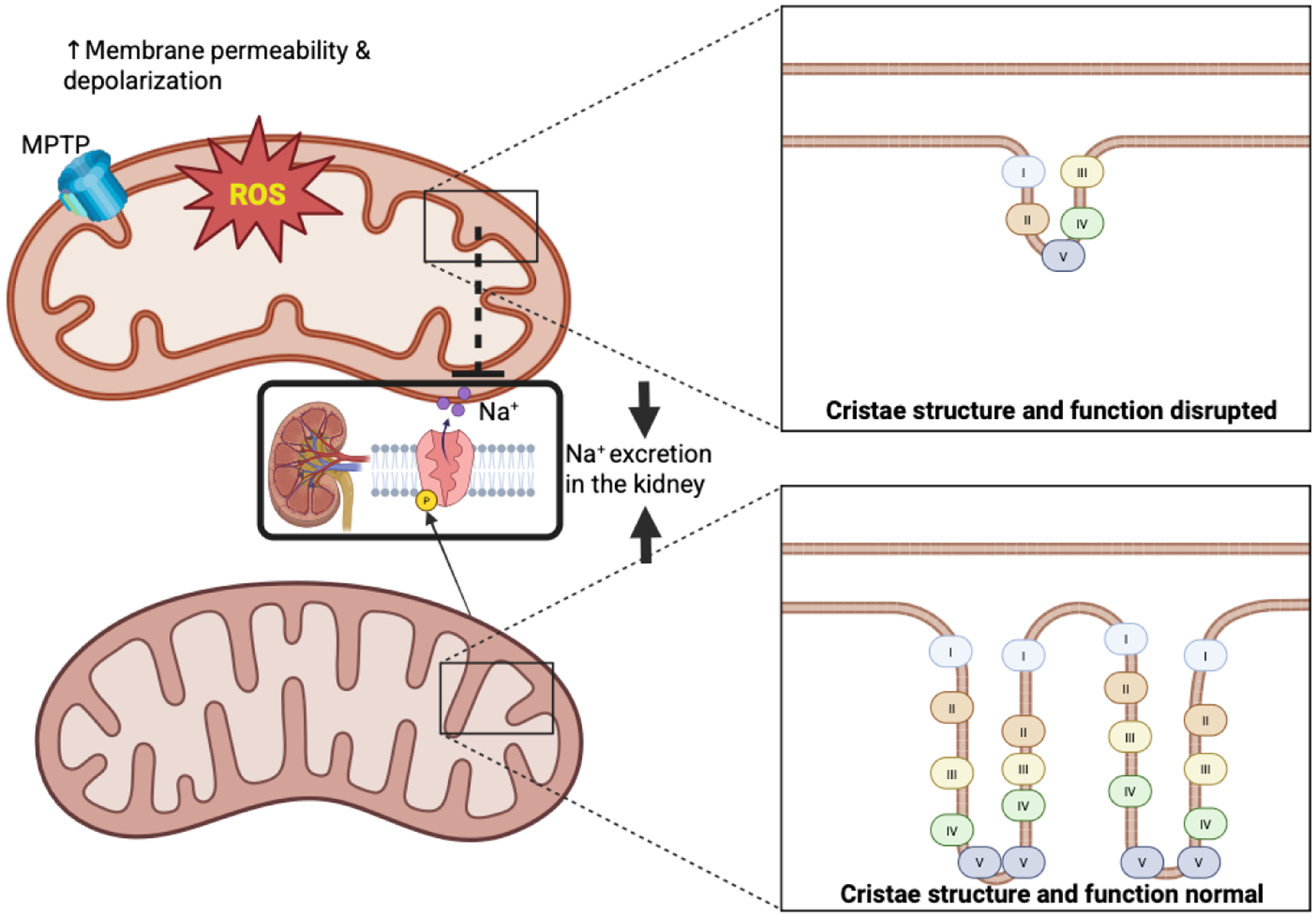
Cristae remodeling and electron transport chain (ETC) dysfunction. An illustration of distorted cristae structure reduced tricarboxylic cycle (TCA) intermediates, impaired electron transport chain (ETC) complexes, and reduced adenosine triphosphate (ATP) synthesis, with arrows linking these defects to sodium retention. Roman numeral boxes represent ETC protein complexes. Created with BioRender.com. MPTP: Mitochondrial permeability transition pore.

Mitochondrial cristae, essential for oxidative phosphorylation, undergo structural changes in SSBP.^60^ Defective cristae remodeling disrupts ETC efficiency, reducing ATP availability for renal sodium excretion (Figure 2). This bioenergetic failure is compounded by leakage of pro-apoptotic factors (e.g., cytochrome c) and increased susceptibility to mitochondrial permeability transition pore (mPTP) opening, promoting cell death in renal and vascular tissues.^61^ Thus, impaired cristae remodeling emerges as a critical driver of bioenergetic failure and cellular injury in salt-sensitive blood pressure, linking mitochondrial structure directly to hypertensive pathology.

### Mitochondrial swelling and membrane integrity loss

High salt intake can lead to osmotic stress and calcium overload, which may trigger mitochondrial swelling and membrane depolarization (Figure 2). This disruption of the mitochondrial membrane potential can impair ATP synthesis and reduce sodium transport efficiency. Furthermore, calcium overload can induce the opening of the mPTP, leading to increased membrane permeability, loss of membrane potential, and diminished ATP production. These mitochondrial dysfunctions contribute to the development of hypertension, especially in salt-sensitive individuals.^60^ Concurrent ROS release and pro-inflammatory signaling exacerbate oxidative stress, endothelial damage, and vascular dysfunction. Notably, salt transiently inhibits mitochondrial energetics in immune cells, contributing to blood pressure elevation.^62^ Altogether, mitochondrial swelling and membrane integrity loss serve as pivotal events in salt-induced hypertension, linking metabolic, vascular, and immune dysfunction through a common mitochondrial pathway.

### Defects in mitochondrial-associated membranes

**Figure 3 |:**
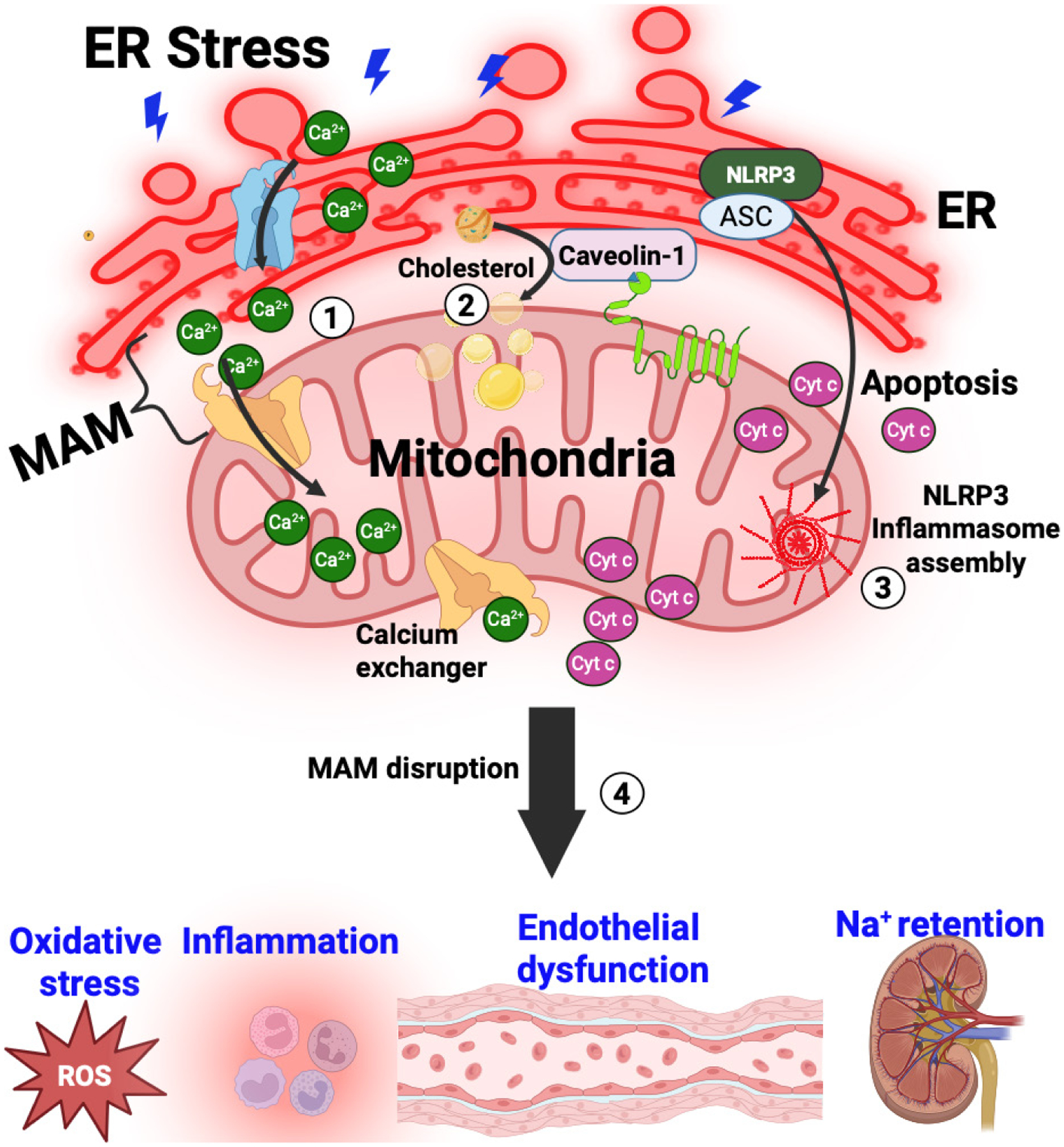
Mitochondria-associated membrane (MAM) disruption in aging. A schematic of disrupted MAMs shows (1) impaired calcium exchange, (2) lipid accumulation, and (3) inflammatory signaling in hypertension. Age-related changes in MAM integrity can disrupt (4) vascular function, increase inflammation and oxidative stress and impair renal sodium handling, raising cardiovascular and renal disease risks. Structural defects in MAM also boost pro-inflammatory cytokine release and metabolic dysfunction, contributing to hypertension. Created with BioRender.com. ASC: Apoptosis-associated speck-like protein containing a CARD; ER: endoplasmic reticulum; NLRP3: NOD-, LRR-, and pyrin domain-containing protein 3; ROS: reactive oxygen species.

MAMs are specialized regions where the ER and mitochondria are closely apposed, facilitating critical cellular processes such as calcium (Ca^2+^) homeostasis and lipid metabolism. These contact sites enable efficient transfer of Ca^2+^ from the ER to mitochondria, which is essential for various metabolic functions. As organisms age, the integrity of MAMs can become compromised, leading to disrupted Ca^2+^ signaling. In VSMCs, such dysregulation may contribute to hypercontractility and structural remodeling, impairing vascular reactivity.^63^ In endothelial cells, altered MAM function has been associated with increased oxidative stress and inflammation, further affecting vascular health.^11^ In the kidneys, MAM dysfunction can disrupt Ca^2+^ signaling pathways that are vital for proper sodium handling. This disruption may lead to impaired renal sodium excretion, contributing to fluid retention and hypertension.^64^ Collectively, age-related alterations in MAM integrity can adversely affect Ca^2+^ homeostasis, vascular function, and renal sodium handling, thereby increasing the risk of cardiovascular and renal diseases (Figure 3). Additionally, structural MAM defects also promote pro-inflammatory cytokine release and metabolic dysfunction, driving hypertension progression.^65^ Together, this MAM-dependent disruption in calcium signaling, metabolic regulation, and inflammatory balance, underscores MAM integrity as a critical determinant of vascular and renal dysfunction in aging-related hypertension.

### Impaired mitophagy and mitochondrial quality control

Mitophagy, the selective removal of damaged mitochondria, is critical for cellular homeostasis. In SSBP, impaired mitophagy leads to the accumulation of dysfunctional mitochondria, perpetuating ROS production and inflammation. Aging exacerbates this by suppressing AMP-activated protein kinase (AMPK)/mammalian target of rapamycin (mTOR) and PINK1-Parkin pathways, reducing mitochondrial turnover. Autophagy dysregulation in renal tissues further compromises sodium homeostasis.^22^ Therefore, impaired mitophagy and disrupted mitochondrial quality control in salt-sensitive blood pressure exacerbate oxidative stress and renal dysfunction, reinforcing the cycle of hypertension and organ damage.

## Underlying Mechanisms Linking Aging and Salt Sensitivity

### Oxidative stress and mitochondrial dysfunction

Aging is marked by a progressive imbalance between mitochondrial ROS production and antioxidant defenses, leading to oxidative damage in vascular and renal tissues.^66,67^ Excess ROS impair nitric oxide bioavailability, promoting endothelial dysfunction and reducing sodium excretion via hyperactivation of renal sodium transporters (e.g., Na^+^/K^+^-ATPase and NCC).^66,67^ This oxidative burden is exacerbated by age-related declines in antioxidant systems such as superoxide dismutase and glutathione peroxidase.^66,67^

### Mechanisms of endoplasmic reticulum stress and mitochondrial crosstalk

**Figure 4 |:**
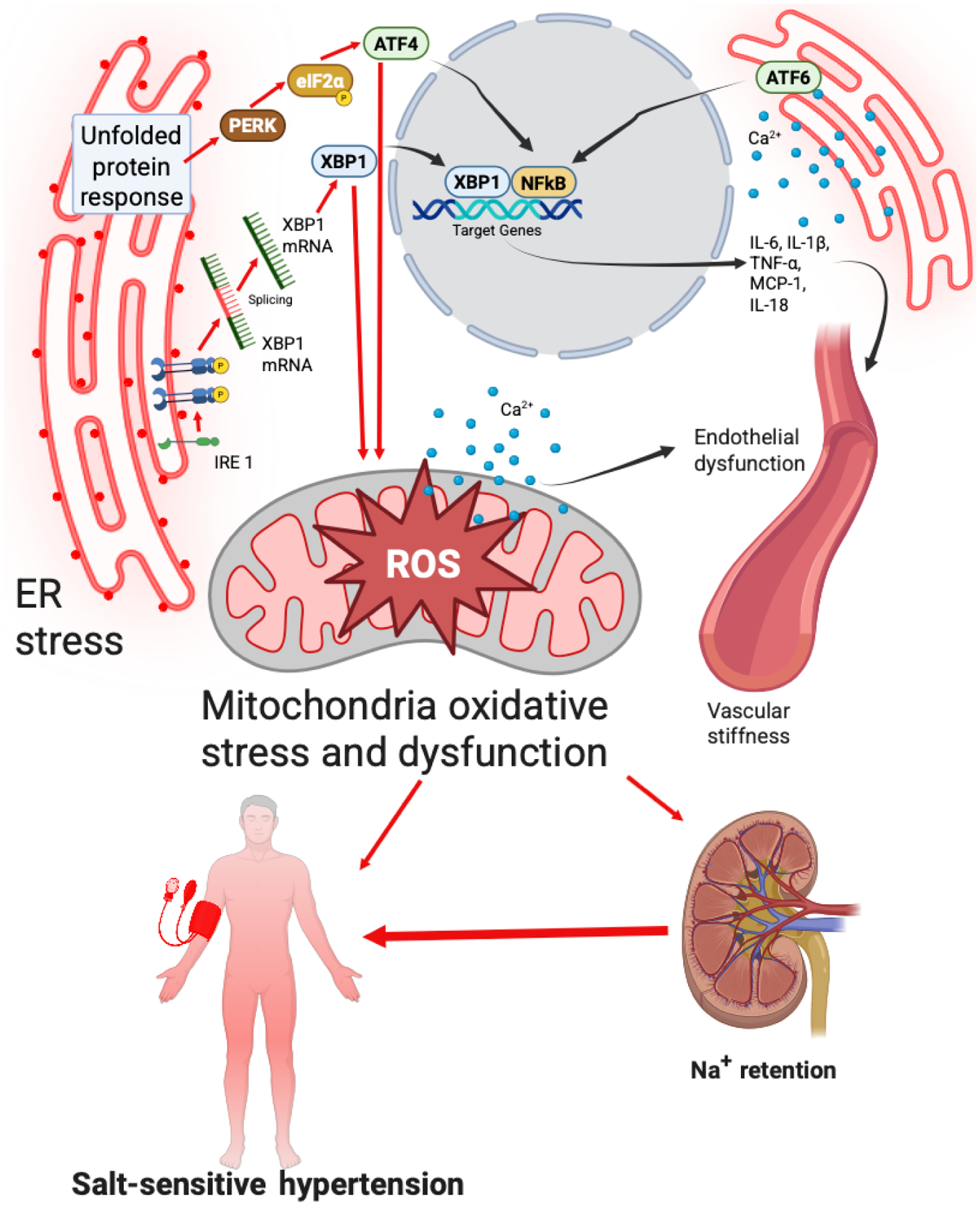
Endoplasmic reticulum (ER)-mitochondria crosstalk in hypertension. An illustration of inositol-requiring enzyme 1 / X-box binding protein 1 (IRE1/XBP1), protein kinase RNA-like endoplasmic reticulum kinase / activating transcription factor 4 (PERK/ATF4), and activating transcription factor 6 (ATF6) transcription and downstream pathways affecting oxidative stress and endothelial dysfunction in hypertension is shown. The unfolded protein response contributes to nuclear factor kappa-light-chain-enhancer of activated B cells (NFκB) transcription, triggering inflammatory pathways. Alongside this, ATF4 and XBP1 result in mitochondrial oxidative stress. Both pathways converge to result in vascular stiffness. Created with BioRender.com. eIF2α: Eukaryotic initiation factor 2 alpha; IL-1β: interleukin 1 beta; interleukin-6; IL-18: interleukin 18; MCP-1: monocyte chemoattractant protein 1; mRNA: messenger ribonucleic acid; TNF-α: tumor necrosis factor-alpha.

The ER and mitochondria interact dynamically through MERCs. Aging disrupts ER proteostasis, triggering the unfolded protein response (UPR) and calcium dysregulation. Chronic ER stress activates three key pathways as follows (Figure 4).

#### Inositol-requiring enzyme 1/X-box binding protein 1 pathway

The inositol-requiring enzyme 1 (IRE1)/X-box binding protein 1 (XBP1) pathway is a crucial component of the unfolded protein response, activated during ER stress. Upon accumulation of misfolded proteins in the ER, IRE1 undergoes oligomerization and autophosphorylation, leading to the splicing of XBP1 mRNA. The spliced XBP1 mRNA is translated into a potent transcription factor that upregulates genes involved in protein folding, secretion, and degradation processes, aiming to restore ER homeostasis. In the context of endothelial dysfunction and hypertension, the IRE1/XBP1 pathway has been implicated in modulating mitochondrial metabolism and inflammatory responses. Activation of this pathway can lead to increased expression of pro-inflammatory cytokines such as interleukin-6 (IL-6) and tumor necrosis factor-alpha (TNF-α), contributing to vascular inflammation and hypertension.^65,68^

#### Phosphorylated protein kinase R-like ER kinase–eukaryotic initiation factor 2 alpha–activating transcription factor 4 pathway

Phosphorylated protein kinase R-like ER kinase (PERK) and eukaryotic initiation factor 2 alpha (eIF2α) enhance ATF4, promoting mitochondrial R O S production and pro-hypertensive inflammation. This pathway links ER stress to vascular stiffness and sodium retention.^65^

#### Activating transcription factor 6 and calcium dyshomeostasis

Activating transcription factor 6 (ATF6) is an ER stress sensor that, upon activation, translocates to the nucleus to regulate gene expression aimed at restoring ER homeostasis. However, chronic activation of ATF6 can disrupt ER-mitochondrial calcium exchange, leading to mitochondrial calcium overload, increased production of ROS, and impaired vascular function.^19^

Together, these ER stress pathways disrupt ER–mitochondrial crosstalk, amplifying oxidative stress, inflammation, and calcium imbalance, thereby contributing to vascular dysfunction and salt-sensitive hypertension.

## Mitochondrial Structural and Functional Alterations in Aging

Mitochondrial biogenesis and dynamics (fusion and fission) are essential for maintaining cellular function.^69,70^ Aging impairs these processes, leading to an accumulation of dysfunctional mitochondria with compromised energy production.^71,72^ Renal epithelial cells and VSMCs exhibit a decline in mitochondrial efficiency with age, increasing susceptibility to salt-induced damage.^73^ Additionally, age-related structural changes in the vasculature, such as increased arterial stiffness, compound salt sensitivity by altering renal perfusion and sodium handling.^74,75^ These changes are closely linked to mitochondrial dysfunction, as impaired ATP production compromises sodium-potassium pump activity, exacerbating sodium retention and volume expansion.

### Mitochondrial dysfunction and oxidative stress in salt-sensitive hypertension

In salt-sensitive hypertension, excessive ROS accumulation exacerbates endothelial dysfunction, renal sodium retention, and vascular stiffness, thereby elevating blood pressure.^19^ Key signaling pathways linking mitochondrial oxidative stress to salt sensitivity include: NADPH oxidase (NOX)-mitochondria crosstalk, mitogen-activated protein kinase pathway, the Nrf2/KEAP1 pathway, and mPTP dysfunction. Relating to NOX-mitochondria crosstalk, aging-associated activation of NOX enzymes enhances superoxide (O_2_^•−^) production, which in turn triggers mitochondrial ROS release, further amplifying oxidative stress.^76^ The activation of p38 mitogen-activated protein kinase and c-Jun N-terminal kinase by mitochondrial ROS promotes pro-inflammatory gene expression and endothelial dysfunction.^12^ Dysregulation of nuclear factor erythroid 2-related factor 2 (Nrf2) signaling impairs antioxidant responses, resulting in unchecked ROS accumulation and endothelial nitric oxide synthase uncoupling. This leads to reduced nitric oxide production and vascular dysfunction, worsening hypertension.^77^ In addition, aging enhances mPTP opening, leading to mitochondrial swelling, cytochrome c release, and apoptotic cell death, which contribute to renal and vascular dysfunction in salt-sensitive hypertension.

Collectively, these interconnected oxidative stress pathways highlight how aging-driven mitochondrial dysfunction amplifies inflammation, endothelial damage, and sodium retention, thereby intensifying salt-sensitive hypertension.

### Mitochondrial-associated endoplasmic reticulum membranes and aging-associated salt sensitivity

MERCs serve as crucial communication hubs between mitochondria and the ER, facilitating calcium exchange, lipid metabolism, and stress responses.^78,79^ In aging, MERC dysfunction disrupts calcium homeostasis, impairing mitochondrial ATP synthesis and increasing ROS production.^80,81^ This imbalance enhances the salt-retentive properties of renal tubules, further driving hypertension. Disruptions in MERCs also contribute to increased inflammatory signaling via the activation of the NLRP3 inflammasome, a pathway implicated in both aging and hypertension.^82–84^ This suggests that interventions targeting MERC integrity could provide novel therapeutic strategies for managing age-related salt sensitivity.

Aging alters mitochondrial morphology and disrupts MERC sites, thereby impairing inter-organelle communication. These changes impact calcium transfer, lipid exchange, and mitochondrial bioenergetics, exacerbating salt-sensitive hypertension.^85,86^ Key molecular regulators include: DRP1, MFN1/2, OPA1, and the VDAC1-IP3R-GRP75 complex. Aging-associated hyperactivation of DRP1 induces excessive mitochondrial fragmentation, reducing mitochondrial efficiency and increasing ROS production. Reduced MFN1/2 and OPA1 expression impairs mitochondrial fusion and cristae remodeling, affecting ATP production and calcium buffering. MERCs regulate calcium transfer via voltage-dependent anion channel 1 (VDAC1) and inositol 1,4,5-trisphosphate receptor (IP3R), mediated by glucose-regulated protein 75 (GRP75).^85,86^ Aging disrupts this complex, leading to mitochondrial calcium overload and hypertension.

## Autophagy, Lysosomal Biogenesis, and Mitochondrial Quality Control

Autophagy and lysosomal degradation pathways are essential for maintaining mitochondrial health by removing damaged organelles.^87^ Aging impairs autophagic flux and lysosomal biogenesis, leading to the accumulation of dysfunctional mitochondria.^88^ In the context of salt sensitivity, defective autophagy exacerbates renal oxidative stress and inflammation, impairing sodium homeostasis.^73,89^ Key regulatory pathways include the AMPK/mTOR pathway, the PINK1-Parkin mitophagy pathway, and the transcription factor EB (TFEB) and lysosomal biogenesis pathway.^73,89^ In the AMPK/mTOR pathway, age-related inhibition of AMPK and activation of mTOR suppresses autophagy, leading to mitochondrial accumulation and dysfunction.^90,91^ With the PINK1-Parkin mitophagy pathway, reduced PTEN-induced putative kinase 1 (PINK1) stabilization impairs Parkin recruitment to damaged mitochondria, leading to defective mitophagy.^92,93^ TFEB is a master regulator of lysosomal biogenesis. Aging-associated TFEB inactivation reduces lysosomal degradation capacity, resulting in mitochondrial and ER stress accumulation.^94,95^

## Mitochondria in Hypertension: the Role of Mitochondrial Contact Site and Cristae Organizing System

### Mitochondrial contact site and cristae organizing system complex

**Figure 5 |:**
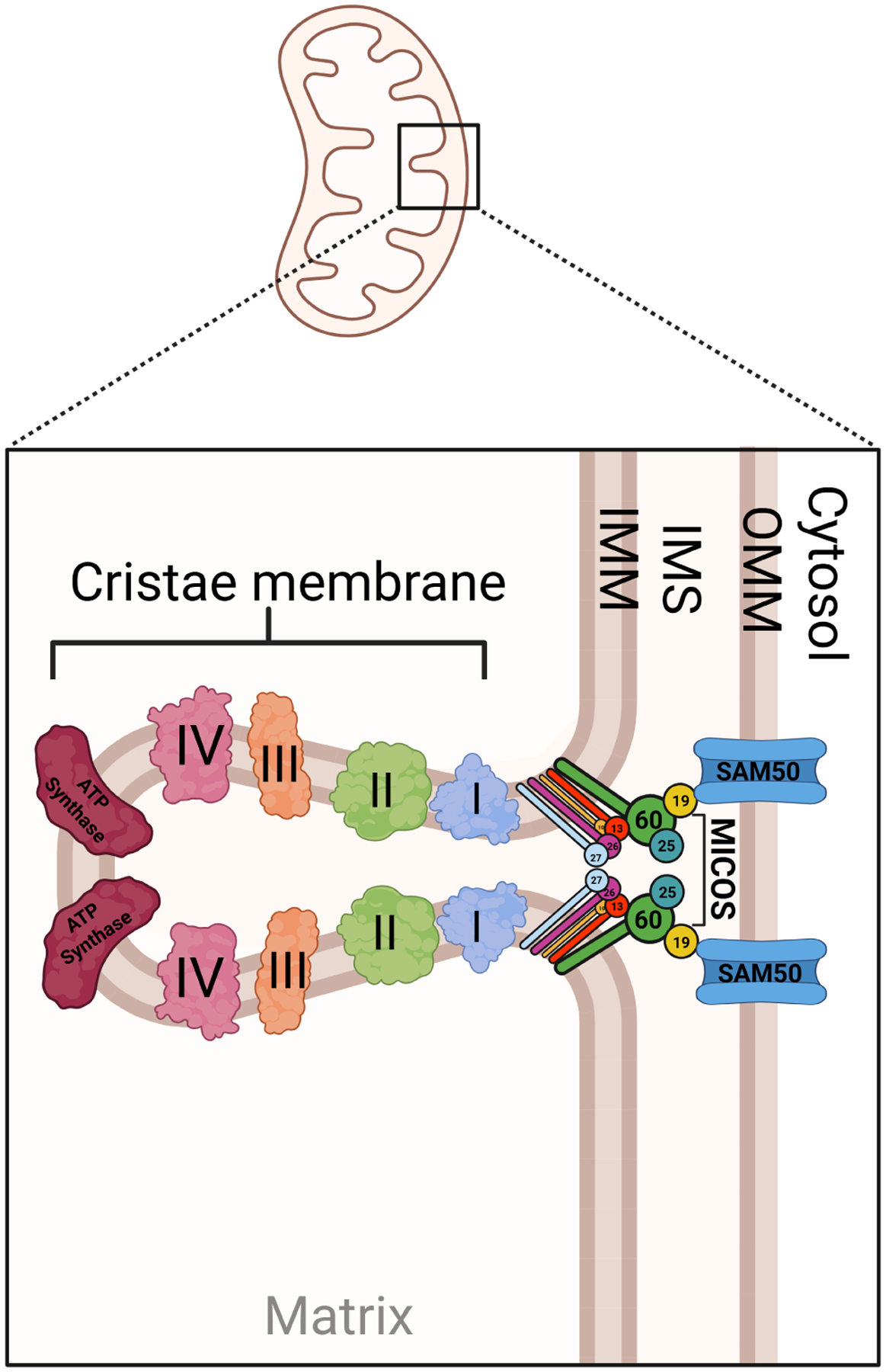
Mitochondrial membranes with mitochondrial contact site and cristae organizing system (MICOS). Depiction of cristae membrane, with numbered MICOS proteins represented in the inner mitochondrial membrane (IMM) and intermembrane space (IMS). On either side, electron transport chain complexes (left) and the channel-forming outer mitochondrial membrane (OMM) protein complex sorting and assembly machinery 50 (SAM50; right) are depicted. Created with BioRender.com.

The MICOS is a multi-subunit complex critical for maintaining cristae junctions and inner mitochondrial membrane architecture (Figure 5).^96^ The mitochondrial cristae are simply inner mitochondrial membrane projections whose structural folds are important for maximizing respiratory capacity.

Mitochondrial inner membranes display remarkable structural diversity across species, tissues, and cell types, forming lamellar sheets, expanded sacs, or various tubular configurations such as round, branched, or prismatic.^97–99^ Energy-intensive tissues (e.g., heart and skeletal muscle) feature tightly packed, thin, parallel cristae, whereas pancreatic cells show broader, more loosely arranged folds.^98,99^ Evidence indicates that each cristae can behave as an autonomous bioenergetic module, maintaining its own membrane potential.^100^ This compartmentalization aligns with observations that respiratory complexes are largely confined within individual cristae, a restriction that likely facilitates mitochondrial quality control by isolating underperforming complexes from fully functional ones.^101^

Composed of core subunits such as MIC60 (mitofilin) and MIC19 (CHCHD3), MICOS ensures proper cristae folding, which is essential for efficient oxidative phosphorylation and mitochondrial ultrastructure.^23^ Cristae junctions mark the narrow portals where the outer membrane, inner boundary membrane, and crista membranes meet.^102^ These neck-like openings are sculpted and stabilized by the multisubunit MICOS.^102^ In mammals, MICOS assembles into two core modules: a MIC60 arm (containing MIC60, MIC19, MIC25) and a MIC10 arm (comprising MIC10, MIC13, MIC26, and MIC27).^28,103,104^ Beyond shaping crista necks, these junctions act as selective gates that slow the lateral spread of proteins and metabolites within the inner membrane, thereby helping preserve the proton gradient and confining ADP/ATP exchange to the respiratory domain.^105^ By anchoring cristae junctions, MICOS facilitates the segregation of inner membrane domains and regulates mitochondrial dynamics, lipid transfer, and apoptotic signaling.^53^ Emerging evidence implicates MICOS dysfunction in the pathogenesis of hypertension and cardiovascular disease, particularly through its role in mitochondrial–nuclear crosstalk and organelle integrity.^106–108^

### Clinical evidence linking mitochondrial contact site and cristae organizing system to hypertension

M I C O S mutations are associated with hypertension in preclinical models. For example, MIC60 knockdown in VSMCs exacerbates ROS production and endothelial dysfunction.^106–108^ Similarly, MIC19 deficiency in renal tubules impairs sodium excretion, promoting salt-sensitive hypertension.^106–108^ These findings highlight MICOS as a potential therapeutic target.

### Visualizing mitochondria

Technological advancements over the past decade, such as focused ion-beam scanning electron microscopy, correlative light-electron microscopy, and 3D tomography, have enabled detailed visualization of mitochondrial morphology in three dimensions during cardiac development and disease progression.^16,53,54,109^ Concurrent innovations, including cryo-electron microscopy (cryo-EM), now resolve oxidative phosphorylation supercomplexes at near-atomic resolution, while high-precision respirometry, proteomic profiling, acetylome mapping, and CRISPR-based gene editing provide deeper insights into mitochondrial function.^110,111^

## Therapeutic Implications: Targeting Mitochondrial Pathways in Aging-Associated Hypertension

Given the intricate role of mitochondria in the pathogenesis of salt-sensitive hypertension, mounting research has turned toward therapies that restore or preserve mitochondrial function in aging individuals. Several promising avenues have been explored as follows:

### Mitochondria-targeted antioxidants

These compounds aim to reduce mitochondrial oxidative stress, a key driver of endothelial dysfunction and renal sodium retention in SSBP. Notable examples include mitochondria-targeted coenzyme Q10 (MitoQ), plastoquinone derivative SkQ1, and elamipretide (SS-31). Preclinical studies in hypertensive models show that such agents can mitigate oxidative damage and improve mitochondrial bioenergetics. In a randomized trial of older adults, oral MitoQ supplementation significantly improved vascular endothelial function and reduced arterial stiffness,^112^ highlighting translational potential for improving age-related vascular health. SS-31, a peptide that targets inner mitochondrial membrane cardiolipin, has advanced to clinical trials in heart failure as reported in a study where long-term SS-31 therapy normalized mitochondrial dynamics and enhanced cardiac energetics in failing hearts.^113^ Moreover, *ex vivo* studies demonstrate that SS-31 can improve bioenergetic function in aged or diseased human myocardium.^114^ These findings underscore that mitochondrial antioxidants are receiving substantial attention, with several compounds (MitoQ and elamipretide) already in human testing for cardiovascular or metabolic indications. While their specific effects on blood pressure await further clinical evaluation, these agents exemplify the therapeutic promise of countering mitochondrial oxidative stress in salt-sensitive hypertension.

### Endoplasmic reticulum stress modulators and mitochondrial–endoplasmic reticulum contact protectors

Because chronic ER stress and disrupted ER–mitochondrial cross-talk contribute to vascular and renal dysfunction in SSBP, chemical chaperones that alleviate ER stress are being explored. Tauroursodeoxycholic acid and 4-phenylbutyric acid (4-PBA) are two such compounds that reduce misfolded protein load and improve proteostasis. In hypertensive animal studies, treatment with tauroursodeoxycholic acid or 4-PBA significantly lowered systolic blood pressure and improved endothelial function.^115^ For example, in spontaneously hypertensive rats, these agents enhanced aortic contractility, reduced vascular oxidative stress, and even attenuated hypertension-related kidney injury.^116^ Notably, 4-PBA treated hypertensive rats showed less proteinuria and glomerular damage, with blood pressure reduction comparable or superior to standard antihypertensive drugs.^117^ Such findings suggest that targeting ER stress and by extension, stabilizing mitochondria–ER interactions at MERCs has a tangible impact on hypertension outcomes in preclinical models. Although tauroursodeoxycholic acid and 4-PBA are not yet used in routine hypertension therapy, their efficacy in models of salt-sensitive or age-related hypertension has prompted interest in repurposing these or related agents. Ongoing research by our group examining whether preserving MERC integrity, for instance, by stabilizing the tethering complexes that regulate calcium flux and ROS transfer can protect against salt-induced blood pressure elevations. This line of inquiry is still in early stages, but it reinforces the concept that safeguarding inter-organelle communication is a viable therapeutic strategy.

### Inhibitors of excessive mitochondrial fission

Aberrant activation of the fission mediator Drp1 in aging and salt overload leads to fragmented, dysfunction-prone mitochondria. Approaches to temper mitochondrial fission have shown positive results in experimental settings. Mdivi-1, a small-molecule Drp1 inhibitor, has been widely used in research to prevent excessive mitochondrial fragmentation.^118^ In a mouse model of angiotensin II–induced hypertension, Mdivi-1 administration attenuated the blood pressure rise by ~22 mmHg and reduced associated arterial remodeling and cardiac hypertrophy.^118^ Likewise, in rodent cardiac stress models, partial inhibition of Drp1 pharmacologically or via genetic means preserved mitochondrial network integrity, improved ATP production, and mitigated hypertensive end-organ damage.^119^ These data suggest that modulating the fission–fusion balance can be beneficial. However, Drp1 inhibitors like Mdivi-1 remain experimental and have off-target effects, so clinical translation will require more specific next-generation compounds or alternative strategies. Nonetheless, the concept of targeting mitochondrial dynamics has gained traction, with ongoing efforts to develop safer fission modulators or gene therapies to boost fusion proteins such as MFN2 or OPA1 in hypertensive organs.^120^

### Autophagy enhancers

Aging is accompanied by a decline in autophagy and mitophagy, leading to the accumulation of defective mitochondria that fuel oxidative stress and inflammation. Thus, stimulating autophagic mitochondrial quality control is an attractive strategy for aging-related hypertension. Several interventions that activate pro-autophagy pathways are under investigation. AMPK activators like metformin and AICAR can induce autophagy and have shown ancillary benefits on blood pressure and vascular function in metabolic syndrome models. More directly, inhibition of mTOR, a nutrient-sensing pathway that suppresses autophagy, has been tested in salt-sensitive hypertension. Notably, chronic low-dose rapamycin therapy in Dahl salt-sensitive rats attenuated salt-induced blood pressure elevation and renal damage.^121,122^ In one study, 3 weeks of rapamycin reduced systolic blood pressure in salt-fed rats from ~176 mmHg to ~153 mmHg.^122^ Similarly, mTORC1 inhibitors like everolimus or the dual mTORC1/2 inhibitor PP242 not only prevented the development of salt-sensitive hypertension but even reversed established high blood pressure in experimental models.^121,123^ These findings highlight the substantial attention on autophagy-related pathways. By restoring the cell’s ability to clear dysfunctional mitochondria and protein aggregates, one can improve mitochondrial function and reduce the pro-hypertensive cellular milieu. While long-term mTOR inhibition in humans has side effects, ongoing research into intermittent dosing or tissue-targeted delivery seeks to harness these benefits safely. In parallel, lifestyle approaches such as caloric restriction and intermittent fasting, which naturally activate autophagy, are being examined for their potential to improve metabolic and blood pressure parameters in older adults.

### Enhancing lysosomal biogenesis (transcription factor EB activation)

An emerging therapeutic angle is boosting the lysosome–autophagy system through TFEB, a master regulator of lysosomal biogenesis and autophagic flux. Preclinical research shows that upregulating TFEB activity can promote the clearance of damaged mitochondria and reduce cellular stress. Compounds such as trehalose, which is a natural disaccharide and resveratrol, a polyphenol, have been identified as TFEB activators.^124^ Trehalose elevates lysosomal biogenesis by inactivating mTORC1 and driving TFEB into the nucleus, thereby enhancing autophagy of dysfunctional proteins and organelles.^125^ Resveratrol likewise has been shown to stimulate TFEB and augment autophagic clearance in models of metabolic and neurodegenerative disease.^124^ Although studies specifically linking TFEB activation to blood pressure control are limited, the concept is gaining interest. By bolstering the cell’s own cleanup and recycling processes, one might curb the mitochondrial ROS and inflammasome activation implicated in salt-sensitive hypertension. Trehalose, for instance, has already demonstrated protective effects in rodent models of kidney injury via autophagy activation,^126^ suggesting relevance to hypertensive nephropathy. Resveratrol, often studied for its cardiovascular benefits, may owe part of its effect to improved organelle quality control in addition to SIRT1 activation. Overall, therapies targeting lysosomal-autophagic pathways are at an earlier stage compared to antioxidants or mTOR inhibitors, but they represent a frontier with considerable therapeutic promise for aging-related diseases, including hypertension.

In summary, a spectrum of mitochondrial-targeted interventions from antioxidants and fission inhibitors to autophagy enhancers and ER stress alleviators has shown efficacy in experimental models of hypertension. Several (such as MitoQ, SS-31, metformin, and rapamycin) have progressed into clinical use or trials for related indications, underscoring growing confidence in this approach. By addressing the mitochondrial and cellular stress dysfunctions at the heart of aging-associated SSBP, these strategies hold the potential to complement traditional blood pressure treatments. Future trials in hypertensive older patients will be critical to determine which of these mitochondrial therapies can translate into meaningful blood pressure reduction or organ protection, and to clarify the optimal timing and combination with standard care.

## Future Directions and Research Gaps

Despite advances in understanding mitochondrial dysfunction in SSBP, key questions remain: How do renal cortical mitochondria specifically contribute to SSBP? What is the precise role of MERCs in aging-associated SSBP? Can mitochondrial-targeted therapeutics be optimized for clinical use?

Additionally, there are limited data on how sex hormones influence mitochondrial responses to salt, as well as gaps in our understanding of renal mitochondrial heterogeneity. The distinct roles of glomerular versus tubular mitochondria in SSBP regulation remain unclear. Furthermore, no large-scale clinical trials have tested mitochondrial-targeted therapies in aging populations. Future research directions should prioritize: MERC-targeted therapies to restore calcium homeostasis via modulation of the VDAC1-IP3R-GRP75 complex, with age, we see a reduction in the MERC complex, contributing to mitochondrial calcium overload and hypertension; multiomics approaches integrating proteomics and metabolomics to map mitochondrial dynamics in aging and precision medicine strategies to develop biomarkers for mitochondrial dysfunction in salt-sensitive hypertensive patients. Addressing these gaps will advance our mechanistic understanding and therapeutic potential for SSBP in aging populations.

## Limitations

While the link between mitochondrial dysfunction and salt-sensitive hypertension in aging is increasingly supported, our current understanding still faces several limitations. First, much of the mechanistic insight derives from animal models or *in vitro* studies and thus, direct evidence in humans remains limited. Rodent models of SSBP such as the Dahl salt-sensitive rat recapitulate many features of human salt-sensitive hypertension, but differences in physiology and the controlled experimental conditions may not fully capture the complexity present in elderly patients with comorbidities.^56^ Clinical studies on SSBP are challenging to conduct due to the lack of an easy diagnostic test, since invasive salt-loading protocols are required for diagnosis of SSBP, this has limited large-scale investigations into how mitochondrial biomarkers correlate with salt sensitivity in diverse populations.^127^ Second, the interplay of aging with sex, genetics, diet, and environmental factors means that dissecting the specific contribution of mitochondrial pathways is difficult. Many older individuals have chronic kidney disease, diabetes, or other conditions that themselves affect mitochondria and blood pressure regulation, confounding the attribution of effects purely to “normal” aging.^128^ Third, although numerous mitochondrial-targeted interventions have shown promise in experimental settings, their translation to clinical therapy faces hurdles. Safety and off-target effects need careful evaluation. For example, long-term mTOR inhibition or global Drp1 inhibition could have deleterious consequences. Moreover, optimal timing and patient selection for such therapies are unknown and whether they would work best as preventive measures in middle age or as treatments in established hypertension is unclear. Finally, this review is limited by the available literature and the scope of our search strategy. It is possible that very recent studies or under-recognized findings were not included. We focused on key pathways (oxidative stress, MERCs, autophagy) known to be relevant, but other emerging mechanisms such as gut microbiome interactions with mitochondrial function were beyond our scope. Despite these limitations, the evidence compiled highlights critical trends and therapeutic hypotheses. Ongoing research employing advanced imaging, omics technologies, and human trials will be essential to fill these knowledge gaps and validate the mitochondrial-centric strategies for combating aging-related salt-sensitive hypertension.

## Conclusion

**Figure 6 |:**
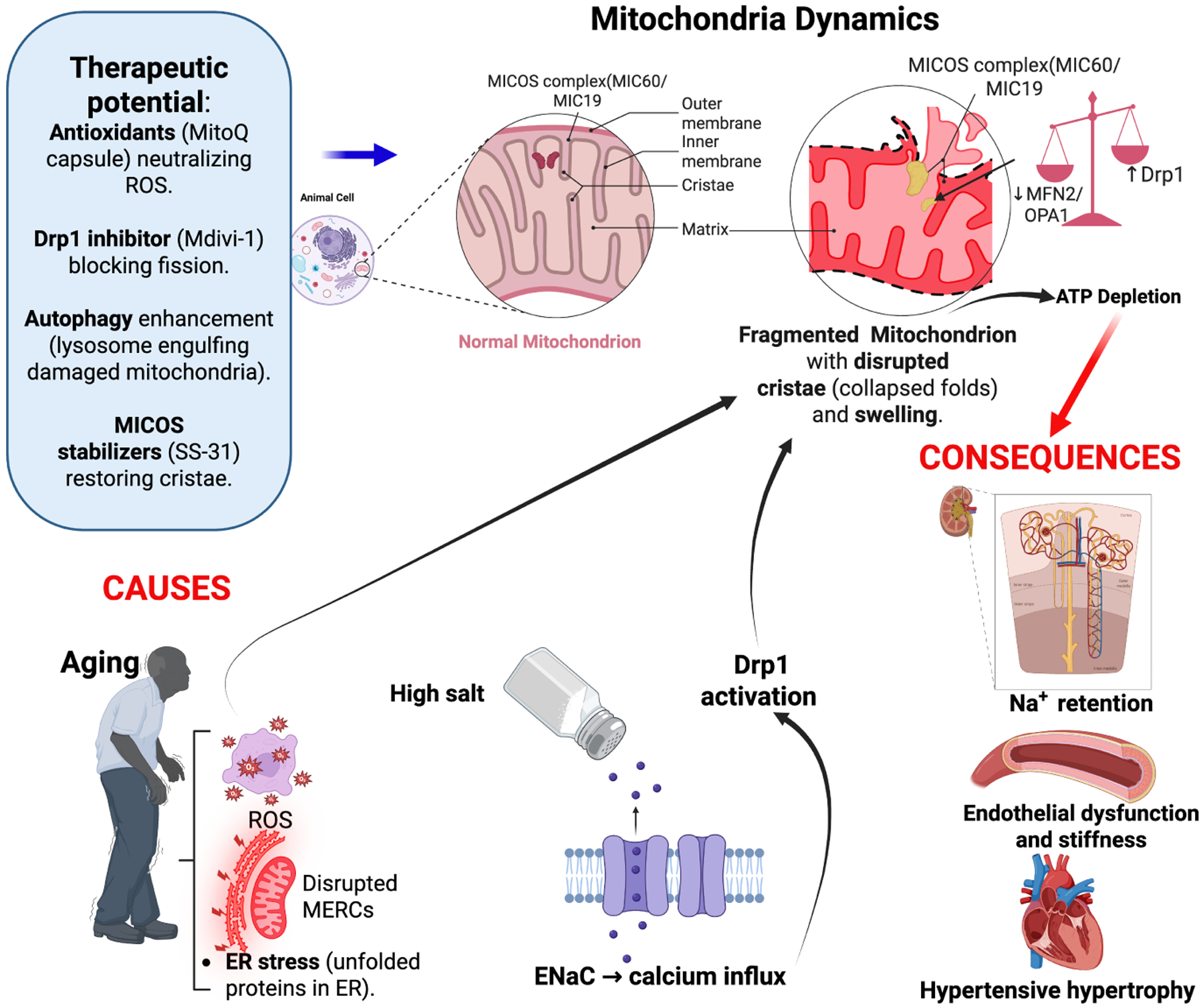
Summary of interplay between aging, mitochondria, and salt sensitive hypertension. Aging and high salt intake disrupt mitochondrial dynamics (fission/fusion), cristae integrity (MICOS), and ER–mitochondria crosstalk (MERCs), driving oxidative stress, impaired sodium excretion, and hypertension. Targeting mitochondrial pathways (e.g., antioxidants, fission inhibitors) restores function. Created with BioRender.com. Drp1: Dynamin-related protein 1; ENaC: epithelial sodium channel; ER: endoplasmic reticulum; Mdivi-1: mitochondrial division inhibitor 1; MERCs: mitochondria–endoplasmic reticulum contact sites; MFN2: mitofusin 2; MIC19: MICOS subunit 19 (CHCHD3); MIC60: MICOS subunit 60 (mitofilin); MICOS: mitochondrial contact site and cristae organizing system; MitoQ: mitochondria-targeted ubiquinone; OPA1: optic atrophy 1; ROS: reactive oxygen species; SS-31: elamipretide.

In this review, we have highlighted the well-established findings showing aging exacerbates SSBP by impairing mitochondrial function, promoting oxidative and ER stress, disrupting MERC communication, and suppressing autophagy. Through these mechanisms, mitochondria contribute to vascular and renal pathophysiology (Figure 6). Thus, targeting mitochondrial signaling pathways represents a promising approach to mitigating salt-sensitive hypertension in the aging population. Future research should explore targeted interventions aimed at restoring mitochondrial integrity, reducing oxidative burden, and enhancing autophagic clearance to improve blood pressure control in aging individuals. Further, focusing on delineating specific mitochondrial-targeted interventions can enhance vascular and renal resilience against sodium-induced blood pressure elevations.

## Figures and Tables

**Table 1 |:** Mitochondrial dynamics in hypertension

Process	Key proteins	Hypertension-associated changes
Fusion	Mitofusin 1/2, optic atrophy 1	Downregulated → Fragmented mitochondria
Fission	Dynamin-related protein 1, mitochondrial fission factor, fission 1 protein	Upregulated → Excessive fragmentation
Biogenesis	PGC1α, NRF1/2, TFAM	Impaired → Reduced mitochondrial mass

PGC1α: Peroxisome proliferator-activated receptor gamma coactivator 1-alpha; TFAM: mitochondrial transcription factor A; NRF1/2: nuclear respiratory factors 1 and 2.
